# Exploring how to enhance care and pathways between the emergency department and integrated youth services for young people with mental health and substance use concerns

**DOI:** 10.1186/s12913-022-07990-8

**Published:** 2022-05-07

**Authors:** Krista Glowacki, Madelyn Whyte, Jade Weinstein, Kirsten Marchand, David Barbic, Frank Scheuermeyer, Steve Mathias, Skye Barbic

**Affiliations:** 1 Department of Occupational Science and Occupational Therapy, The University of British Columbia, Faculty of Medicine, T325 – 2211 Wesbrook Mall, Vancouver, BC V6T 2B5 Canada; 2 Foundry Central Office, Providence Health Care, 1881 Burrard, Vancouver, BC V5G 7H9 Canada; 3 Centre for Health Evaluation & Outcome Sciences, Providence Health Care, #588-1081 Burrard Street, Vancouver, BC V6Z 1Y6 Canada; 4 Providence Health Care Research Institute 1081 Burrard Street, Vancouver, BC V6Z 1Y6 Canada; 5 Department of Emergency Medicine, St Paul’s Hospital and the University of British Columbia, 1081 Burrard St, Vancouver, BC V6Y 1Z6 Canada; 6 Faculty of Medicine, University of British Columbia, Vancouver, British Columbia Canada; 7 Department of Psychiatry, University of British Columbia, Vancouver, British Columbia Canada; 8 Department of Psychiatry, Providence Health Care, St. Paul’s Hospital, Vancouver, British Columbia Canada

**Keywords:** Integrated youth services, Emergency department, Mental health, Substance use, Young people

## Abstract

**Background:**

Integrated youth services (IYS) provide multidisciplinary care (including mental, physical, and social) prioritizing the needs of young people and their families. Despite a significant rise in emergency department (ED) visits by young Canadians with mental health and substance use (MHSU) concerns over the last decade, there remains a profound disconnect between EDs and MHSU integrated youth services. The first objective of this study was to better understand the assessment, treatment, and referral of young people (ages 12–24 years) presenting to the ED with MHSU concerns. The second objective was to explore how to improve the transition from the ED to IYS for young people with MHSU concerns.

**Methods:**

We conducted semi-structured one-on-one video and phone interviews with stakeholders in British Columbia, Canada in the summer of 2020. Snowball sampling was utilized, and participants (*n =* 26) were reached, including ED physicians (*n =* 6), social workers (*n =* 4), nurses (*n =* 2), an occupational therapist (*n =* 1); a counselor (*n =* 1); staff/leadership in IYS organizations (*n =* 4); mental health/family workers (*n =* 3); peer support workers (*n =* 2), and parents (*n =* 3). A thematic analysis (TA) was conducted using a deductive and inductive approach conceptually guided by the Social Ecological Model.

**Results:**

We identified three overarching themes, and factors to consider at all levels of the Social Ecological Model. At the interpersonal level inadequate communication between ED staff and young people affected overall care and contributed to negative experiences. At the organizational level, we identified considerations for assessments and the ED and the hospital (wait times, staffing issues, and the physical space). At the community level, the environment of IYS and other community services were important including wait times and hours of operation. Policy level factors identified include inadequate communication between services (e.g., different charting systems and documentation).

**Conclusions:**

This study provides insight into important long-term systemic issues and more immediate factors that need to be addressed to improve the delivery of care for young people with MHSU challenges. This research supports intervention development and implementation in the ED for young people with MHSU concerns.

## Background

Symptoms of mental health and substance use (MHSU) challenges start early in life, with 50–70% developing during childhood or adolescence [1]. The peak age of onset for MHSU disorders is 14.5 years (k = 14, median = 18, interquartile range (IQR) = 11–34) [2]. In Canada, 20% of youth have a mental disorder [1], and one in four young people (ages 12–24) experience a MHSU disorder each year, with most presenting to the emergency department (ED) as their first point of contact for health services [3, 4]. According to recent surveillance data from the Canadian Institute for Health Information (CIHI) between 2008 to 2019, there was a 61% rise in mental health related ED visits by Canadian children and youth [1]. Evidence also suggests that more than half of Canadian youth and young adults presenting to the ED with MHSU needs have not had any previous mental health related-contact with the health-care system [5, 6]. In BC, the opioid use epidemic, declared a public health emergency in 2016, has compounded the MHSU concerns for young people, with youth and young adults accounting for 20–25% of all opioid-related overdoses and deaths [7]. In two other Canadian provinces (Ontario and Alberta), young people aged 15–24 had the highest rates of ED visits for opioid poisoning from 2010 to 2011 and 2014–2015 [8]. Exacerbating these trends, there is currently a profound disconnect between the ED and MHSU services for young people. Young people are presenting to the ED as their first point of contact for MHSU concerns but due to various reasons may not be admitted to the hospital [4]. Thus, it is necessary that ED staff are equipped to refer and connect such young people upon presentation to the appropriate MHSU services for care and follow-up.

Integrated youth services (IYS) are one example of a community-based, youth- and family-centred MHSU service. Integrated youth services provide multidisciplinary care (including mental, physical and social aspects), and are preferably in one location that is a youth-friendly environment [9, 10]. Young people and their families and needs are prioritized in design of services and care [11]. IYS exist internationally, and Hetrick and colleagues provide a detailed overview of development and evaluation from a global perspective [9]. Examples of IYS internationally include headspace™ in Australia [12], Jigsaw in Ireland [13], Irish Youth One Stop Shops (YOSS) [14], Les Maisons des Adolescents in France [15], and Youth One Stop Shops (YOSS) in New Zealand [16]. IYS have also been established in Canada; however, they are more infant in their development: Youth Wellness Hubs Ontario (YWHO) [17], and Foundry [11, 17].

IYS have existed for over two decades (the earliest IYS was established in New Zealand in 1993) [16]. Pathways to such services (and other mental health care for young people) are complex, involve multiple contacts and can have delays to getting appropriate care [18]. Important areas of care pathways to consider include primary care and the roles of families. In addition, the high rate of emergency services involvement in such pathways makes the care pathway between the ED and mental health services for young people necessary to consider [18]. Little is known about referral pathways for young people between the ED and IYS offering MHSU support. There is an immediate need to improve referrals to organizations that can provide ongoing services for MHSU concerns in order to deliver appropriate care and prevent repeat ED visits and hospitalization for MHSU disorders. There were two main objectives of this study: (1) to better understand the assessment, treatment, and referral for young people (ages 12–24 years) presenting to the ED with MHSU concerns; and (2) to explore how to improve the transition from the ED to IYS for young people with MHSU concerns.

## Methods

### Setting

This study took place in British Columbia (BC), Canada. In the province of BC, Foundry is an integrated youth service (IYS) in the community that provides care (including MHSU services) for young people ages 12–24 years [11]. There are currently eleven centres across BC, with twelve more in development, and a provincial virtual care service [19]. Foundry has five core service streams, including mental health, substance use, peer support, primary care, and social services.

### Approach

Since little is known about the transition from the ED to IYS for young people with MHSU concerns in Canada, we conducted a qualitative study for exploratory purposes. The Standards for Reporting Qualitative Research (SRQR) was referenced to guide study reporting [20].

### Participants

Ethical approval was obtained from the University of British Columbia Ethics Board (H20–01396). To be eligible for participation, individuals had to live in the province of BC and self-identify as one of three stakeholder groups: (1) health care providers with experience in the ED (e.g., nurses, physicians) or experience working with individuals with MHSU disorders in any setting; (2) individuals with experience working for an agency or institution in any capacity focused on young people with MHSU concerns; and/or IYS (3) young people (age 16–24) and/or family members of young people with personal lived experience with MHSU concerns.

Participants were recruited from across the province of BC, Canada, through purposive snowball sampling. The authors FS DB and SM are health care providers working in ED and IYS settings. All reached out to colleagues that met eligibility requirements to begin this process. The aim was to recruit diverse stakeholders from a variety of communities considering population centre density, their local services, and communities with and without IYS in the community.

### Data collection and procedures

Semi-structured one-on-one video (over the platform Zoom Video Corporation, San Jose, CA) or telephone interviews were conducted by MW, JW, SB, and FS. MW and JW were research assistants at the time of the study. Data collection occurred June–August 2020 with rolling admission into the study. Participants provided verbal or written consent at the beginning of the interview after reviewing an information sheet about the study. As part of this process, participants were informed that all questions were optional and that they could end the interview at any time. The semi-structured interview (Table 1) addressed the care experiences of young people accessing the ED for MHSU concerns, including assessments used and treatments provided. Questions also focused on the transition and referral pathways from the ED to IYS in the community such as Foundry, and vice versa. Lastly, considerations were given to what an ideal process would look like for the transition between the ED and services in the community, as well as improving young people’s experiences while in the ED. Following the interview, participants were asked five optional demographic questions about age, gender, race, role/position, years in practice, and community of practice as applicable. Participants were provided a $40 gift card for participating.

**Table 1:** Interview Script

Introduction	Hello, my name is XXX, I am a member of the research team affiliated with this project. Thank you for participating. The following questions will take us approximately 30 minutes to complete. If you do not wish to answer the question, please say pass.
**Interview Script**	1) Describe the current experience of a young person, aged 12–24 years, accessing the emergency department in your community for mental health or substance use challenges. 2) A. Describe the experience of a young person from the point they walk through the ED doors to the point where they’re discharged? B. Can you describe the current assessments that you know are available in the ED for young people with mental health or substance use challenges? 3) What would you recommend as an ideal assessment process for this population? 4) Describe what happens to a young person once they have been identified for discharge to the community? 5) Can you describe what you know about Foundry services in your community? 6) What would you recommend is an ideal process to connect young people to Foundry from the ED? 7) Last question, if a young person presents to Foundry, and needs ED services, what do you recommend is an ideal process to connect young people from Foundry to the ED?
**Optional Demographic Questions**	Please identify the following: age, gender, race/ethnicity. What community do you live in? Are you a student, where do you go to school? What is your area of practice? Years in practice?

### Data analysis and theory

**Fig. 1:**
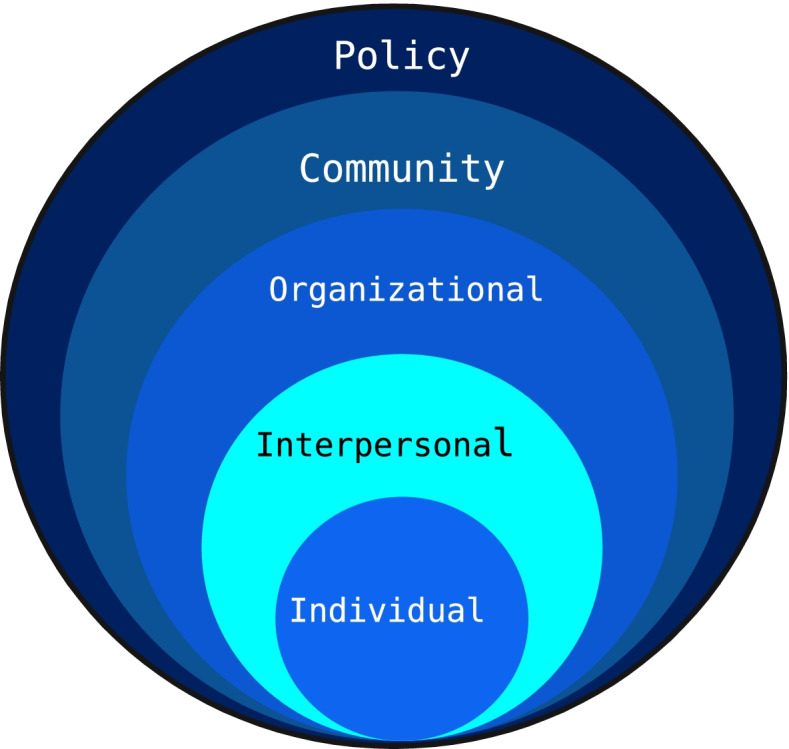
Social Ecological Model (adapted from Bronfenbrenner, 1979)

All interviews were audio-recorded and transcribed verbatim. To maintain confidentiality, unique participant numbers were assigned and all potentially identifying information was removed from the transcripts. Analysis was conceptually guided by the Social Ecological Model (SEM) [21]. The SEM is a framework that helps to understand how individuals interact with their environment within a complex social system and considers various connections to an individual [21]. It considers and acknowledges that important determinants to health exist at multiple levels, including that of the individual, interpersonal, organizational, community and policy (Fig. 1). The SEM has been used previously and acknowledged as helpful for understanding suicide assessment and prevention [22], for transitions of young people within health care services [23], and acknowledged as important in considering experiences in an ED after opioid overdose [24]. The SEM has also been recommended when considering transformation of traditional silos and acute treatment in health care, acknowledging it is necessary to consider social determinants of health for individuals [25]. The research team considered the individual a young person with MHSU concerns and determined definitions of all levels of the SEM relevant to the research objectives to guide analysis (Table 2).

**Table 2:** Social Ecological Model Definitions developed by the research team

SEM Level	Definition
Individual level	A young person with MHSU concerns. Factors at this level were considered internal to the individual including but not limited to biological, personal history, age, education, income, beliefs, symptoms, coping skills.
Interpersonal level	Relationships and social connections to a young person with MHSU concerns. Factors considered were relationships (with family members, health care providers, peer support workers), family support/lack of support, household living situations, social connections (or lack of) with peers/peer support workers/health care providers.
Organizational level	The emergency department within the hospital. Examples of factors considered were rules and regulations; hours; resources such as beds, staffing, etc. While focus was on the ED, the larger institution of the hospital and the ED within it were also considered.
Community level	IYS services in the community such as Foundry, and other services for young people with MHSU concerns. Factors here included the transition from the ED/hospital, access to the community services (post-discharge from the ED), as well as the care pathway from the community back to the ED.
Policy level	Local, provincial, and national laws/regulations relevant to the health and health care of a young person with MHSU concerns. Examples of factors considered were charting systems throughout the province, bills, provincial health coverage.

A deductive and inductive analytical approach was used in a multi-step process to conduct a thematic analysis [26, 27]. NVivo 12 Software (QSR International, Doncaster, AU) was used to manage data and support qualitative data analysis. KG & MW engaged in active analysis through several phases to ensure thoroughness [27]. The phases included becoming familiar with the data—listening to audio recordings and reading transcripts while notes were taken. Next, codes were generated in NVivo based on the coding framework created using the SEM and definitions. This step was considered deductive, and coding was conducted by KG & MW independently. Regular meetings were held to ensure understanding of the codes and concept definitions and KG and MW coded one transcript together during this time. The broader codes were then reviewed to search for and identify themes inductively. This entailed searching through each level of the SEM codes to find data-driven ideas that were commonly occurring relevant to the research question (and ultimately developed into themes). Themes were then reviewed across and within the entire data set (and within and across SEM levels) and discussed by KG & MW to stimulate dialogue and encourage researcher reflexive acknowledgment of results and perspectives in the research process [28]. Themes were then defined and named. Writing of themes was an iterative and an integrated process throughout to aim for a comprehensive analysis. To aim for quality thematic analysis, the 15-point “checklist” for good thematic analysis was consulted [26].

### Findings

#### Characteristics of study participants

The sample included 26 individuals with a mean age (±SD) of 42 (±12.3). A variety of stakeholders participated including ED physicians (*n =* 6), social workers (*n =* 4), nurses (*n =* 2), an occupational therapist (*n =* 1); a counselor (*n =* 1); staff/leadership in IYS organizations (*n =* 4); mental health/family workers (*n =* 3); peer support workers (*n =* 2), and parents (*n =* 3). For service providers, the average number of years in practice (±SD) was 14 (±7.4). The majority identified as women (19/26; 73%) and white/Caucasian (20/26; 78%). Participants were from 9 different communities with over half (*n =* 15) being from a medium sized urban population centre as defined by Statistics Canada [29]. Further details of participant characteristics are provided in Table 3. Demographic questions were open ended and so responses to questions vary (e.g., race/ethnicity identity).

**Table 3:** Participant Characteristics

Characteristic of Participants (***n =*** 26)	
**Age in years** M (SD)	42 (12.3)
**Range**	26–60
**Designation** % (N)	
ED physician	22 (6)
Social worker	15 (4)
Nurse	8 (2)
Occupational therapist	4 (1)
Counselor	4 (1)
Staff/leadership in IYS organization	15 (4)
Mental health/family worker	12 (3)
Peer support worker	8 (2)
Parent	12 (3)
**Number of years in practice** M (SD)	14 (7.4)
**Range**	0.75–30
**Gender** % (n)	
Women	73 (19)
Men	19 (5)
Non-binary	4 (1)
Not disclosed or other	4 (1)
**Race** % (n)	
White/Caucasian	77 (20)
Indigenous	11 (3)
Asian	8 (2)
European	4 (1)
**Rural/Urban Centre % (n)**	
**Urban centre (population size)**	
Small population centre (1000-29,999)	Total = 8 (2)
Cowichan, BC	4 (1)
William’s Lake, BC	4 (1)
Medium population centre (30,000-99,999)	Total = 57 (15)
North Vancouver, BC	23 (6)
Prince George, BC	15 (4)
Victoria, BC	11 (3)
New Westminster, BC	4 (1)
Pencticton, BC	4 (1)
Large population centre (> 100,000)	Total = 31 (8)
Vancouver, BC	23 (6)
Kelowna, BC	8 (2)
Unknown	4 (1)

#### Findings of the thematic analysis

**Fig. 2:**
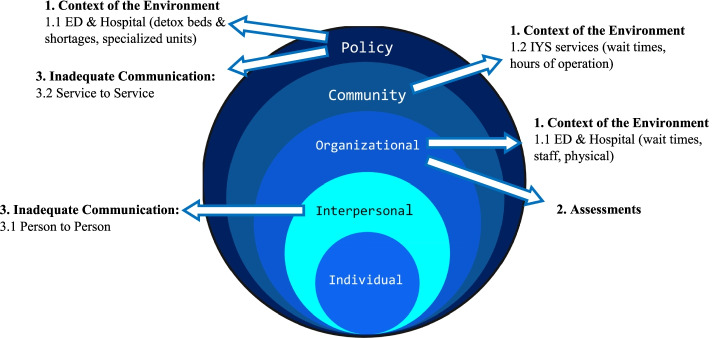
Results of thematic analysis and the Social Ecological Model

The themes and sub-themes are discussed in detail below and outlined in Fig. 2 in relation to the Social Ecological Model.

### Theme 1 Context of the Environment

Participants discussed the impact that the ED and hospital contexts have on young people’s transitions from ED to IYS, with a focus on staff schedules, hours of operation, wait times, and the physical environment.

### Environment of the Emergency Department and Hospital

**Fig. 3:**
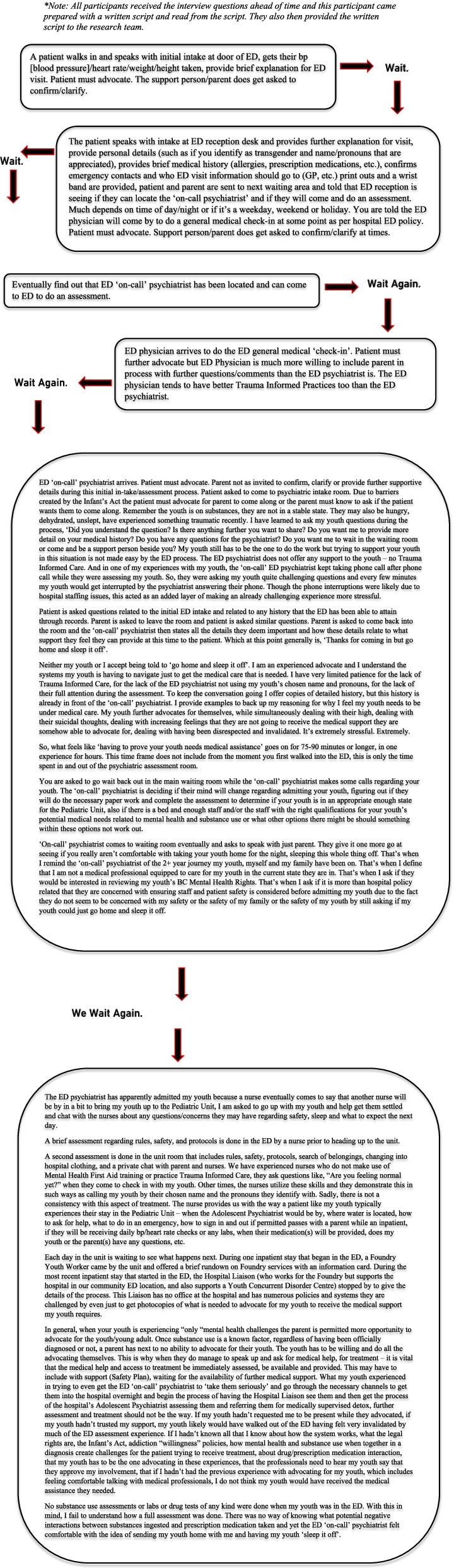
A Parent’s story of waiting with their young person at the ED.*. *Note: All participants received the interview questions ahead of time and this participant came prepared with a written script and read from the script. They also then provided the written script to the research team

Within the ED, participants discussed that time played a significant role in the experience of a young person presenting with MHSU concerns. This was in regard to the timing of MHSU designated staff shifts in the ED, the amount of time waiting and how it was spent while waiting in the ED to be assessed, admitted or discharged. Participants expressed concern for how long a young person waits in the ED when they are in a crisis and presenting in distress. For instance, participants spoke of this experience taking up 12 hours or days, and that health status may significantly change in this time. Participants acknowledged that the wait times varied based on available staff in the ED and demand at time of presentation. They also suggested improving the wait time experience by providing care packages to increase comfort while young people wait, with items such as a water bottle, a snack, a blanket, and an activity to do. Figure 3 provides a vignette of the perspective of a parent waiting in the ED for their child to receive care. Another participant shared their experience:

“In the ER is it’s still a lot of time before someone really sits down and runs you through an assessment like that and for some of the youth that I have supported in going to the ER that length of time means that they’re actually fine now so they kind of get stuck in a system where they’re like people still want to talk to them and don’t really want to let them go but **at this point like no one has talked to them for 12 hours so what’s the point in being there anymore.**” *IYS Staff, Participant #4*

Another participant expressed a similar concern and said, “There’s often long wait periods, historically I’ve experienced with young people where they’ve been left in ER hallways for hours and sometimes days.” (*Early Intervention Assertive Case Manager*, *Participant #15*).

In addition to wait times, participants spoke of the timing of staff shifts in the ED, which could hinder or enhance a young person’s experience. For example, when young people present to the ED “after hours” there are usually less staff, resulting in higher wait times. Participants also spoke of specialized MHSU staff in the ED, including child psychiatrists and registered nurses with an addiction or mental health specialty. Although these service providers were seen to enhance care, participants noted that their hours of work were limited (e.g., Monday-Friday during the daytime) and so weren’t always available when needed. These aspects of time and resources (wait times and staff shifts) were considered at the organization level of the SEM. A parent highlighted this issue:

“The really big problem is that often he [young person] would go to the Emergency Department at the end of the week and then these, **the addiction team isn’t available on the weekends** **or the evenings** so instead of keeping him over the weekend so he could see someone, they just discharged him onto the street, like he can’t stay with us because of his issues and because of my daughter so he just goes back onto the street, it’s pretty sad.”

*Parent, Participant #17.*

Another participant acknowledged the limited resources in the ED and their experience:

“When I was a young person my experience in the ER was a life changing one for me but had more to do with the other lived experience in the ER than it did for any of the staff that were there. **I think I could really tell that the staff were super burnt out, like they didn’t have the resources that they want and that kind of left them not able to listen or hear the stories of people who were there with them** but for me the like being able to connect with other people who have lived experience that was there really life changing thing.”

*IYS Staff, Participant #4.*

Participants also spoke about the physical environment of hospital EDs and the impact this could have on young people. Within the ED, participants expressed concern over poor lighting, lack of private spaces or rooms while waiting, and congestion of people making it “quite loud and busy” (*Early Intervention Assertive Case Manager, Participant 15*). These were considered organization level factors of the SEM. Participants also discussed that there are a limited number of beds within the ED and for admission to a certain ward (psychiatric wards and pediatric wards were discussed). In addition, depending on age or certain MHSU needs, admission to either of these units may be inappropriate. Participants also spoke of a lack of youth-oriented substance use treatments available (e.g. detox beds, pharmacological and psychosocial treatments) in a hospital and in the community. Bed shortages and units/wards were considered policy level factors of the SEM. One participant discussed both the environment in the ED and admission to a unit:

“You’ve got multiple nurses coming and going in the next thing you know security guards are coming in and all kinds of stuff so you know you’re just kind of whatever **you’re just sort of pushed off to the side, they don’t have the capacity to deal with it and it’s not a nice patient to deal with right**?

… By the time they stabilized, they’re ready to get out of there because they are in a pediatrics unit so it’s already odd because you know they’re next to a child who may have a broken leg or you know a baby that’s in there for a broken arm, it’s a mixed bag. There really isn’t any place for them”.

*IYS Leadership, Participant #23.*

#### Environment of Integrated Youth Services

Participants were concerned regarding the hours of IYS availability and access to specialists (such as a psychiatrist). Generally, community IYS are open from 8:30 am-4:30 pm on weekdays; if a young person is discharged from the ED at any other time, they are left to initiate IYS contact themselves. These were considered community level factors of the SEM. One participant discussed the repercussions of the limited IYS hours and connection to ED discharges:

“This young person just showed up and we told them to come to Foundry, if there had been more ongoing care then there might be some discharge planning and some connections that are available and built but kind of quite often young people and their families are just left to navigate that in some ways on their own … you know **I know it’s more challenging that we’re [IYS] kind of a Monday-Friday 9–5 industry and they’re a 24-hours a day, 7 days a week kind of industry, right?** So how do we do that on a Saturday evening after a young person has overdosed or maybe showed up because you know they’re at risk of harm …

This really came up for us over the last year and a half, we’ve had, we had sort of a period of you know 3 young people who had died by suicide and then within a couple months another couple people you know just really realizing that you know sort of those bigger players that interact with young people like mental health and substance use services, education services and others really don’t know what’s happening on the weekends, right?”

*IYS Leadership, Participant #7.*

### Theme 2 Assessments

Participants that worked in the ED spoke of not knowing how to assess for MHSU concerns and using MHSU-related assessments, especially for young people. Standardized scoring systems and needing to get a certain score to show a level of crisis through reported symptoms were also discussed. Lastly, participants discussed current assessments used and recommendations for improvement. All factors about assessments in the ED were considered the organizational level of the SEM.

### Not for young people

Participants were unclear with regard to youth-appropriate ED assessments and resources and described a lack of confidence and knowledge when assessing or referring young people with MHSU challenges. In response to being asked about assessments a physician responded:

“Well there are certainly different scoring systems that we use, scoring system that we use for alcohol intoxication, COWS [The Clinical Opiate Withdrawal Scale] is a scoring system that we’ll use for alcohol overdose or opioid withdrawal symptoms. I know that there are several agitation scores that do exist, we don’t often or certainly I don’t use those I don’t have a numeric scoring system that I use for agitation but I do know those exist. **I don’t think there are any specific scoring systems for mental health issues or formalized assessments systems.**”

*Interviewer “*How comfortable are physicians saying stay/go for young people with mental health issues?”

“I would say uncomfortable.”

*Interviewer “*How often do you think the physicians make the decisions themselves?”

“I would say often enough because we see such a high volume of substance abuse and addictions or mental health excuse me that we can’t refer everyone to psychiatry so we sort of have a process where we definitely will refer who we think are higher risk and the younger the person the more likely we are to refer and** the comfort level is lower with younger patients.**”

*ED Physician, Participant #24.*

### That Magical Number

Participants spoke about young people knowing that when they were screened or assessed as a patient in the ED that a certain score or cut-off point was needed to either be admitted or get referred to services. They explained that they would use this knowledge to downplay symptoms if they wanted to go home, or on the other hand identify symptoms they knew would get them help. One participant explained their experience with this:

“People kind of knew that the ER was assessing you and so there became this idea that people, if you want help then you have to make yourself look as bad as possible and make yourself** show as many symptoms as possible so that you would meet that magical number. **I think whatever assessment people are gonna use the 5 or 10 points before you hit that magical number or even that magical range also suck … I know that every assessment has young people who don’t think it’s that hard to fill out and there are people who fucking hate it so it’s kind of hard to pick one in particular but for me it’s more just what we do before **what we do with the people who don’t meet the criteria and how we can give them better resources ‘cause they’re clearly also not doing well, no one wants to waste their evening in a hospital.**”

*IYS staff, Participant #4.*

Physicians in the ED corroborated this, explaining that assessments and screening are used to determine if a young person is in crisis and the level of severity to determine if they should see a mental health specialist (such as a psychiatrist) for an evaluation. A physician explained this process from their perspective:

“I’d say at least two-thirds of the kids we see actually do not require an emergent psychiatric assessment and in fact just needs some directions as to what the situation is, get some perspective on where, what kind of resources exist for their needs and then **I talk to them about the various resources and the way I talk about three tiers, **like the highest tier is the most emergent they need to see a psychiatrist really for the perspective of hospitalizing them, to keep them safe and stabilize until we can connect them to community resources. The second tier is when I will put in place a crisis response team and crisis type management whereby a psychiatrist or a mental health team provider will contact them the next day or so to see what the situation is and help them navigate the healthcare system and the mental health resources.”

*ED Physician, Participant #9.*

### The Many Aspects of Health

Participants identified specific assessments used in the ED (see Table 4 for further details) and acknowledged that ED assessments were inconsistent, especially regarding substance use. They encouraged recognizing factors beyond self-harm risk and incorporating a holistic approach to consider mental, physical, and sexual health, substance use, social determinants of health (such as housing and social supports) and culture. A parent shared her story of her experience with her child in the ED with regards to assessments, as well as recommendations for improvement:

“I keep coming back to a substance use assessment done by a trained and experienced professional … A medical professional suggesting that a parent take a youth home who is high, drunk, suffering from suicidal ideation, taking prescription medication, soon to be coming down off their high – this is highly dangerous in my mind and experience. **If the medical professionals and the parent have no clear idea of what they are trying to support or possibly will need to manage than how can anything other than having the youth stay in hospital **where potential necessary treatment is immediately available even be considered, let alone suggested …

A substance use assessment may include questions and observations related to identifying where challenges are existing and how problematic these challenges are. These areas may include but are not limited to family life, social experiences, school/work life, mental health state, changes in behaviour, appearance, coordination (walking, sitting, writing), acute and chronic memory issues, red or glazed eyes, dilated or constricted pupils, unusual body odors, tremors, under-weight, low energy, bruising, dehydrated, dizzy, sleep related issues, etc.”

*Parent, Participant #26.*

**Table 4:** Specific Assessments identified by participants used in the ED

Participant Quote	Full Name of Assessment	Reference
HEARTSMAP	–	[30]
HEADS-ED	–	[31]
COWS	The Clinical Opiate Withdrawal Scale	[32]
TASR-aM	Tool for Assessment of Suicide Risk: Adolescent Version Modified	[33]
IS PATH WARM	Mnemonic for risk factors: Suicide Ideation, Substance Abuse, Purposeless, Anger, Hopelessness, Trapped, Withdrawing, Anxiety, Recklessness, Mood	[34]

### Theme 3 Inadequate Communication

Participants emphasized young people having to repeat painful or traumatic histories to multiple care providers and having providers deliver inconsistent or incorrect information when referring to other services. All communication between a young person and another person was considered at the interpersonal level of the SEM. In addition, communication between various services (different charting systems and medical records) made it a challenge for continuity of care for young people. The inconsistency and inability of charting systems to communicate across the province was considered a policy level factor.

### Person to Person

The direct communication between health care providers and young people had a significant impact on a young person seeking help. A recurring concept discussed was that when young people present to an ED with a MHSU concern, they are in a very vulnerable state where they want or need help. They are then asked to *repeat* their story numerous times to each care provider throughout this process, which can be a difficult and traumatic experience. A participant expressed their frustration with young people having to repeat stories:

“If at all possible if folks could read the story, rather than having a young person repeat it over and over and over again so there is sort of this description where you come in, go through intake, you tell your story, you see the nurse, you tell your story, you see somebody else a liaison or anybody else, you tell your story, you see the doctor, you tell your story, if you get to a specialist, you tell your story, so there’s testimony is **telling stories you know like 5, sometimes 6 times to different individuals along the way which was really exhausting and problematic.**”

*Family Services Project Developer, Participant 22.*

In addition to repeating stories to different people, participants cited negative experiences when young people were not treated as a person first during conversations with care providers. This left youth in the ED feeling invalidated, unsafe, and stigmatized during an already traumatic experience. Participants acknowledged the importance of the communication with health care providers, as one negative experience can keep a young person from seeking out help again in the future. A participant shared their experience:

“I went in [to the ED] and I was sharing the bit, struggling with eating disorder and I had someone respond “so you just want to be skinny, that’s your issue”?... it just felt icky to me or I’ve had times where my lab work was very off and **I had a nurse say to me “don’t you realize you’re killing yourself here, like this is very serious, do you not see how serious this is”? And again it wasn’t in a compassionate light**, I’ve had friends who had struggled more with I think like bulimia and when they opened up about their binging/purging there was this kind of horrified look on the physician’s face and they shared with me how they just felt so embarrassed that they even admitted to what they were doing. **I think sometimes we hope as people who’ve struggled when we share these things be met with this empathy and understanding.**”

*Peer Support Worker, Participant #4.*

A parent also shared their experience with a child in the ED and the “lack of trauma-informed care”. She summarized her experience and compared the difficulties of mental health compared to physical health:

“So, in general the experience of the process of acquiring support through the ED is quite **stressful, unsupportive, difficult to navigate and not all validating**. It is not as simple as going in and saying you need medical assistance and then being supportively brought through the appropriate steps of care, the way which you would if you went in for a broken limb or a high fever with severe nausea and emesis.”

*Parent, Participant #26.*

Participants also spoke about miscommunication regarding referrals from the ED to community IYS based on personal conversations of what they were told in the ED. This then led to unrealistic expectations upon referral with regard to wait times for services and what services are offered. A participant discussed the miscommunication about referral to other services:

“So I think for me it’s less the assessment and more like really encouraging people to do to like give those like good resources and not be lazy about it, I think there sometimes **people who will give out a resource but they don’t know anything about how to access that resource, they don’t know if that resource has space the number of young people.** I know [people] who’ve gotten a resource from the ER after being told they weren’t going to get a bed for that night and then they call that place and there’s a 3-year waiting list.”

*IYS staff, Participant #4.*

Participants also spoke about receiving pamphlets as a referral with no explanation beyond this which was not helpful communication. A parent summarized their experience:

“**We just get pamphlets and the pamphlets aren’t helpful** … For my son, when he’s discharged, he’s handed from PED [pediatrics] he’s handed a bunch of pamphlets maybe a packet of pills that he just gobbles down, **he’s feeling rather hopeless, helpless**, he’s given a bunch of numbers to call, if that, which he doesn’t, he’s not able to he’s just not able to, he had gone there for help but yeah wasn’t able to get any.”

*Parent, Participant #17.*

### Service to Service

Participants identified communication when transitioning between services to be suboptimal, as there is a lack of communication between charting systems in different agencies (even within the same province). This again led to repetition of young people’s stories (as discussed above in ‘Person to Person’ communication). Participants acknowledged the need for continuity of care through improved charting systems and referrals. A physician shared this from their perspective:

“It’s just a hope you say ‘go to Foundry!’ **But no part of their chart, nothing follows them to there** so they can go to the Foundry and Foundry may have no idea that they’ve even been to the emergency department or what their concerns were at the time or why they’re even showing up.”

*ED Physician, Participant #1.*

Another health care provider discussed the difficulty with systems communicating and highlighted this, “continuative care just gets totally dropped, the only way it survives is if we’re all on the same medical record so that’s something that kind of keeps that continuative care alive” (*Social Worker, Participant #5).*

## Discussion

Through our study and thematic analysis, we identified important factors at all levels of the Social Ecological Model (SEM) that need to be considered for young people with MHSU concerns presenting to the ED and referral to other community-based services such as IYS. While factors were identified at the individual level, these results could not be presented since no participants were considered the age of a young person (12–24 years) at the time of the study. At the interpersonal level, inadequate communication between ED staff and young people affected overall care and contributed to negative experiences when seeking help. Other studies in Canada have found that individuals of all ages presenting to the ED with MHSU concerns reported feeling stigmatized and discriminated against by staff in the hospital based on their communications with providers [35, 36]. Hawk and colleagues acknowledged the importance of interpersonal relationships and the need for sympathetic health care staff in the ED after an overdose [24]. A systematic scoping review identified therapeutic alliance and health care staff showing empathy and non-judgement as being of high importance for individuals accessing substance use disorder treatment [37]. In Canada, recovery-oriented guidelines exist for mental health practice that recommend use of similar principles such as recognizing a person first, affirming autonomy, focusing on strengths, and building collaborative relationships [38]. However, Canadian emergency physicians have very limited formal MHSU training, often less than a few weeks in a single setting. Our results indicate that training for ED clinicians on MHSU, recovery principles, and trauma-informed care could improve the care for young people. Purkey and colleagues have also acknowledged the need for training on trauma-informed care within the ED, as well as the need for institutional buy-in and have outlined detailed recommendations to do so [35].

At the organizational level, context of the environment was identified as important, with considerations for assessments, the ED and the hospital (specifically wait times, staffing issues, and the physical space). Regarding assessments, participants expressed a need for them to be youth-friendly, to be appropriate for MHSU concerns, and to consider health beyond symptoms and scoring. Within the ED in Canada, there is little guidance and great variation with respect to how to assess and treat young people presenting with MHSU concerns [30]. Two mental health assessment tools for young people have been developed in Canada: HEARTSMAP [31] and HEADS-ED [39]. Tools acknowledged for use of substance use assessment are the CRAFFT tool, which considers a variety of substances; AUDIT/AUDIT-C, which considers alcohol use disorders; and two questions on the NIAA tool [40, 41]. However, participants acknowledged that youth-oriented assessments that do exist for MHSU concerns are not mandated or regulated in Canada; thus, use of them varies depending on the clinician. No guidelines or consensus statements from national organizations such as Canadian Association of Emergency Physicians or National Emergency Nurses Association exist. Newton and colleagues have also acknowledged that specialized instruments to screen for and diagnose MHSU problems for young people are not yet standard components of clinical assessments in the ED [42]. While various assessments exist for different reasons, our results indicate that the development of a comprehensive biopsychosocial assessment that is youth-friendly and considers mental health concerns, substance use, and other important social determinants of health such as housing and social supports would be highly beneficial for both ED clinicians and young people. Such an assessment would also need to be standardized for physicians working with this population in that it should be used consistently across EDs and IYS across the province (with findings shared with consent and referral) to enhance continuity of care and transitions between services.

Wait times and the experience while waiting was a concern identified in this study. This could be due to limited resources such as staffing shortages and other care priorities, especially during the global COVID-19 pandemic [43]. It is likely that at the time of the study priority in hospitals was put on those presenting with COVID-19. However, most of our participants who spoke of lived ED experience referenced this from the past and likely prior to the pandemic. Participants spoke of the benefit of having psychiatric nurses in the ED setting to potentially address some of these concerns. Work has been done within Canada to better understand this role, and patients, families as well as ED staff in general valued such an addition in the ED [44]. However, such specialized staff vary in Canada and are only embedded within certain hospitals. Thus, without adequate resources (such as specialized MHSU staff including psychiatric nurses) in the ED, it warrants consideration of ED staff capacity to do thorough assessment, treatment, and referral in a youth and person-centered way. One of our participants spoke of their personal lived experience and acknowledged seeing burned-out staff in the ED. Since EDs may be the only access point for care of young people with MHSU concerns after IYS hours of operation and in communities without IYS or MHSU services, hospitals and staff need to be better equipped to deal with young people presenting with concerns. One consideration is the implementation of mental health staff within EDs. This could include peer support workers who may utilize their own lived experience to aid in people presenting with concerns. A study has been done to explore the potential in Australia acknowledging the important skills peer support workers could bring [45]. Also in Australia, a new model of mental health liaison nursing care has been implemented and evaluated within EDs [46–48]. Some of the key guiding principles of this model include a lead nurse practitioner/consultant to a designated team of specialist mental health nurses during extended hours 7 days per week to work closely with other ED staff and complement psychiatry services [46]. Initial evaluation of this is promising, showing a reduction in ED length of stay, less wait times to see a professional, referrals to community mental health teams, and clinician and patient acceptability [46]. Patients acknowledged the therapeutic benefits including being listened to and understood [47, 48]. Implementation of such a model in Canada would require significant resources and organizational change. However, as our results indicate, the EDs are currently significantly under resourced, especially for people presenting with MHSU concerns. The authors conducting this work in Australia acknowledge the need for resources, structural considerations within the ED, and connections to other mental health services [47] which would all be necessary considerations for a similar model to be used within Canada.

With regard to the community level, the context of the environment of IYS and other community services were identified as important. Specifically, this included wait times and hours of operation. Important policy level factors identified include inadequate communication between services (e.g., different charting systems and documentation) and context of the environment when considering the ED and hospital—specifically shortages of detox beds and specialized units for young people with MHSU concerns. Systemic changes to address these concerns include a universal charting system within the province, more detox facilities or designated detox spaces embedded within the hospital, and perhaps specialized MHSU units for young people.

### Future directions

The results indicate there are systemic and long-term policy changes needed including development of a MHSU assessment for young people, specialized units, detox beds, increased staffing, and some aspects of the physical environment (e.g., paint, lighting, chairs). However, the results also indicate that there are certain areas of improvement to be addressed immediately. This includes communication between health care providers and young people, the experiences of young people waiting in the ED, and potential implementation of a combination of existing mental health, substance use, and suicide risk assessments. The working team (authors) for this project (ED2Foundry) has received funding to implement a small-scale intervention in two EDs in BC to enhance the experience of young people with MHSU concerns and referral to IYS in the community such as Foundry. The team is currently using the results from this study to consult with young people and ED staff to develop, implement, and evaluate an intervention.

### Strengths and limitations

The study had a wide range of stakeholders with lived experience; however, not having the firsthand perspective of young people at the time of the study was a limitation. The authors attempted to enroll young people as participants and asked all participants if they could share study details with their networks. The study team was contacted by one young person who participated in an interview; however, the interview was inaudible, and the data could not be used. This limited contact could be perhaps due to this group having increased MHSU concerns actively at the time of the study and not being able to participate. This study occurred in the early stages of the COVID-19 global pandemic. In attempt to reconcile this, the team worked to obtain the perspectives of young people through parents, who shared both their own experiences and their child’s experiences, and current adults shared their own MHSU experiences from when they were young. For health care providers (other than the ED physicians and IYS staff) not all disclosed whether they worked in the ED or the community when demographic data was collected thus the authors could not tease this apart when interpreting results. The study was guided by a theoretical framework [21]. Several steps were taken by the authors described in the data analysis section to ensure rigour [28]. The authors also consulted the 15-point “checklist” for good thematic analysis [26]. The authors are currently discussing the results with youth with lived/living MHSU experience to determine the future intervention aspect.

## Conclusion

This study identifies important factors to improve the ED care experience of young people presenting with MHSU concerns and referral to appropriate services such as IYS. This includes long-term systemic changes such as the development of a MHSU assessment for young people and aspects of the hospital and ED environment such as increased staffing, specialized units and staffing and the physical space. The results also indicate areas of improvement to be addressed immediately, such as communication between health care providers and young people, and the current wait time experiences of young people while in the ED when resources are limited. This study supports intervention in the ED for young people presenting with MHSU concerns and the authors are using the results of this study to inform intervention development and implementation.

## Data Availability

The datasets generated and analyzed during the current study are summarized in this manuscript. The raw data are not publicly available due to the possibility of confidentiality of participants being compromised. Contact the corresponding author krista.glowacki@ubc.ca should you have any questions about access to data.

## Abbreviations

IYS: Integrated youth services
ED: Emergency department
MHSU: Mental health and substance use
BC: British Columbia
SEM: Social Ecological Model
